# Novel Gel Formulation and Deep Injection Techniques for Lifting Effects in Cosmetic Dermatology

**DOI:** 10.1111/jocd.16789

**Published:** 2025-01-10

**Authors:** Irina Poleva

**Affiliations:** ^1^ CGH Compagnie Generale des Hopitaux Rome Italy

**Keywords:** amino acids, lifting effect, poypeptides

## Abstract

**Introduction:**

In recent years, the field of aesthetic dermatology has witnessed a surge in demand for minimally invasive procedures aimed at rejuvenating aging skin. This study aims to address this demand by evaluating the effectiveness of the injectable gel in rejuvenating aging skin, particularly by targeting collagen regeneration and lifting effect.

**Materials and Methods:**

The study involved 43 participants who underwent three monthly injection sessions targeting retaining ligaments. The injections were administered deeply to ensure proper targeting. Follow‐up assessments were conducted after each treatment session and three months after the final injection. Evaluation methods included subjective assessments by both patients and investigators using the Global Aesthetic Improvement Scale (GAIS), as well as objective assessments using a 3D photosystem to measure wrinkle conditions and vectors of traction.

**Results:**

All participants completed the study, with no significant adverse effects observed apart from mild swelling at the injection sites. Despite the high viscosity of HA necessitating the use of a 27 G needle, the injection process was generally comfortable and minimally painful. Subjective evaluations revealed consistent improvements in skin appearance from the first application, which continued to increase throughout the study and remained high even 3 months post‐treatment. Objective evaluations demonstrated significant improvements in wrinkle conditions and lifting effects, with a substantial increase in the standard deviation score for wrinkle conditions and the average traction vector length measuring 1.6 mm.

**Conclusion:**

The study findings confirm the safety and efficacy of the injectable formula, with high patient satisfaction, noticeable lifting effects, and significant improvements in wrinkle conditions. These results support the use of the injectable as a promising option for non‐invasive skin rejuvenation treatments.

## 1 Introduction

In recent years, the field of aesthetic dermatology has witnessed a surge in demand for minimally invasive procedures aimed at rejuvenating aging skin. This burgeoning interest can be attributed to several factors, including advancements in medical technology, increased awareness of personal appearance, and the aging of the global population. As individuals seek ways to maintain a youthful and vibrant appearance, the demand for safe, effective, and non‐invasive treatments has become increasingly pronounced [1, 2].

Aging is a natural biological process characterized by a gradual decline in the structural integrity and functional capacity of various bodily systems, including the skin. One of the most visible manifestations of aging is the progressive loss of skin elasticity, firmness, and hydration, accompanied by the formation of wrinkles, fine lines, and sagging skin. These changes are primarily attributed to intrinsic factors, such as genetic predisposition and hormonal fluctuations, as well as extrinsic factors, including cumulative sun exposure, environmental pollutants, and lifestyle habits such as smoking and poor nutrition [3]. From this perspective, the aging of the subcutaneous connective tissue, including the ligaments, plays a crucial role [4].

Traditionally, invasive procedures such as facelifts, chemical peels, and laser resurfacing have been the primary modalities for addressing age‐related skin concerns. While these techniques can yield significant improvements in skin appearance, they are often associated with inherent risks, prolonged recovery times, and the potential for adverse effects such as scarring, infection, and pigmentary changes. Moreover, the invasive nature of these procedures may deter individuals who prefer less invasive interventions or who are apprehensive about undergoing surgery. In response to these challenges, there has been growing interest in the development of non‐invasive techniques for skin rejuvenation that offer comparable efficacy with minimal downtime and reduced risk of complications.

Among these, injectable formulation has emerged as a promising modality for delivering bioactive compounds directly into the dermis and subcutaneous tissue, where they can exert their therapeutic effects on key cellular components involved in skin aging. This formula is meticulously engineered, incorporating a tailored ensemble of amino acids (glycine, proline, lysine, alanine, valine, arginine, and leucine), selected polypeptides (acetyl decapeptide 3, oligopeptide 24, acetyl tetrapeptide 5), and a blend of high and low molecular weight hyaluronic acid (HA), each chosen for its strategic role in the preservation of youthful skin integrity and the biochemical pathways that govern aging.

The strategic inclusion of amino acids like glycine, proline, and lysine, which are predominant in the triple helix structure of collagen, directly supplements the skin's most abundant protein, acting as building blocks for tenoblasts and fibroblasts, promoting the synthesis and organization of new collagen fibrils [5]. The presence of alanine, valine, arginine, and leucine, vital to the skin's defense system, supports cellular defense mechanisms and engages in the regulation of tissue repair and regeneration [6].

The polypeptides, namely acetyl decapeptide 3, oligopeptide 24, and acetyl tetrapeptide 5, function as biomimetic messengers that emulate the activity of growth factors, stimulating the dermal fibroblasts to produce more collagen and elastin [7]. These peptides have been recognized for their ability to enhance skin elasticity and density, offering a promising solution to the structural compromise that accompanies the aging process [8, 9].

Finally, the blend of high and low molecular weight HA provides high viscosity for mechanical support though non‐crosslinked, maintains deep hydration, volume, and turgor. The larger molecular size of HA is instrumental in forming a stable, long‐lasting matrix within the dermal interstitium, offering not only immediate plumping effects but also sustained rejuvenation [10, 11]. Moreover, the anti‐inflammatory properties of high molecular weight HA contribute to the reduction of oxidative stress within the skin, supporting a healing and restorative environment [12].

Low molecular weight HA (LMW‐HA) has emerged as an important molecule with distinct biological activities conducive to skin rejuvenation [13]. Low molecular weight HA, achieved through either enzymatic or mechanical breakdown of its high molecular weight counterpart, offers benefits beyond mere moisture retention. It penetrates the epidermis more effectively due to its smaller size, facilitating a range of skin‐enhancing outcomes. Scientific evidence suggests that LMW‐HA can induce a variety of responses from the skin cells, including the promotion of angiogenesis, modulation of inflammatory processes, and stimulation of collagen synthesis [14] Moreover, LMW‐HA's ability to modulate inflammation is pivotal. It can attract immune cells to injury sites, promoting a controlled inflammatory response that is necessary for effective tissue repair [15]. This property can be especially beneficial following aesthetic procedures that trigger an inflammatory response as part of the skin's natural healing process.

Therefore, this injectable blend transcends the mere concept of skin rejuvenation targeting the subcutaneous connective tissue and ligaments, where aging and environmental factors can significantly impair structural integrity and skin appearance by aiming at promoting collagen production in these deep connective tissue. It is a sophisticated complex, thoughtfully composed to synergistically fortify the connective tissue biomechanical properties. The formulation encapsulates a holistic strategy to counteract the aging process, substantiating its potential as a transformative force in cosmetic dermatology [16].

This innovative gel is designed to be injected deeply targeting the retaining ligaments. Subcutaneous connective tissue, comprising fibrous bands and adipose deposits, acts as a support structure for the skin. The degradation of this tissue contributes to the visible signs of aging, such as sagging and the loss of facial contours. The introduction of this gel into the subcutaneous layer is intended to replenish the extracellular matrix, thus providing mechanical support and biological signals as well as collagen building blocks that encourage the regeneration of the connective tissue.

Similarly, the ligaments in the facial structure, which anchor the skin to underlying bone, are also subject to age‐related changes, leading to laxity and a shift in facial dynamics. By targeting these deeper structures, the gel acts on the foundational elements of facial architecture, offering a tangible lifting effect [17]. The strategic delivery of this gel to such depth ensures that the active ingredients can exercise their regenerative potential where it is most needed, effectively restoring the supportive function of ligaments and enhancing the integrity of the subcutaneous connective tissue.

This approach follows the principle of treating the underlying causes of skin laxity, rather than merely the symptoms, offering a more enduring solution to the challenges of skin aging [18, 19].

## 2 Materials and Methods

### 2.1 Study Design

This study was designed as a retrospective, single‐center clinical trial to evaluate the efficacy and safety of a novel injectable gel composed of essential amino acids, polypeptides, and high and low molecular weight HA, for non‐invasive skin rejuvenation (Jalupro Superhydro, Professional Derma, Swiss). The trial was conducted in accordance with the principles of the Declaration of Helsinki. All participants provided written informed consent prior to enrollment.

### 2.2 Participants

A total of 43 (1 male, 42 female patients) healthy adult volunteers aged 35–65 (mean 51.02) years with visible signs of facial aging, including wrinkles, fine lines, and skin laxity, were recruited for the study. Inclusion criteria included good general health, no history of hypersensitivity to components of the gel, and a willingness to comply with study procedures and follow‐up visits. Exclusion criteria included pregnancy, breastfeeding, active dermatologic conditions, and prior facial cosmetic treatments within the past 6 months.

### 2.3 Injectable Gel Composition

The injectable gel formulation was meticulously engineered, incorporating:

- Amino acids: glycine, proline, lysine, alanine, valine, arginine, and leucine, essential for collagen synthesis, and skin repair.
- Polypeptides: acetyl decapeptide 3, oligopeptide 24, and acetyl tetrapeptide 5, known for their ability to stimulate collagen, and elastin production.
- High viscosity complex: High molecular weight HA (> 2000 kDa) and low molecular weight HA (50 kDa) were used to provide mechanical support and deep hydration.

### 2.4 Procedure

**FIGURE 1:**
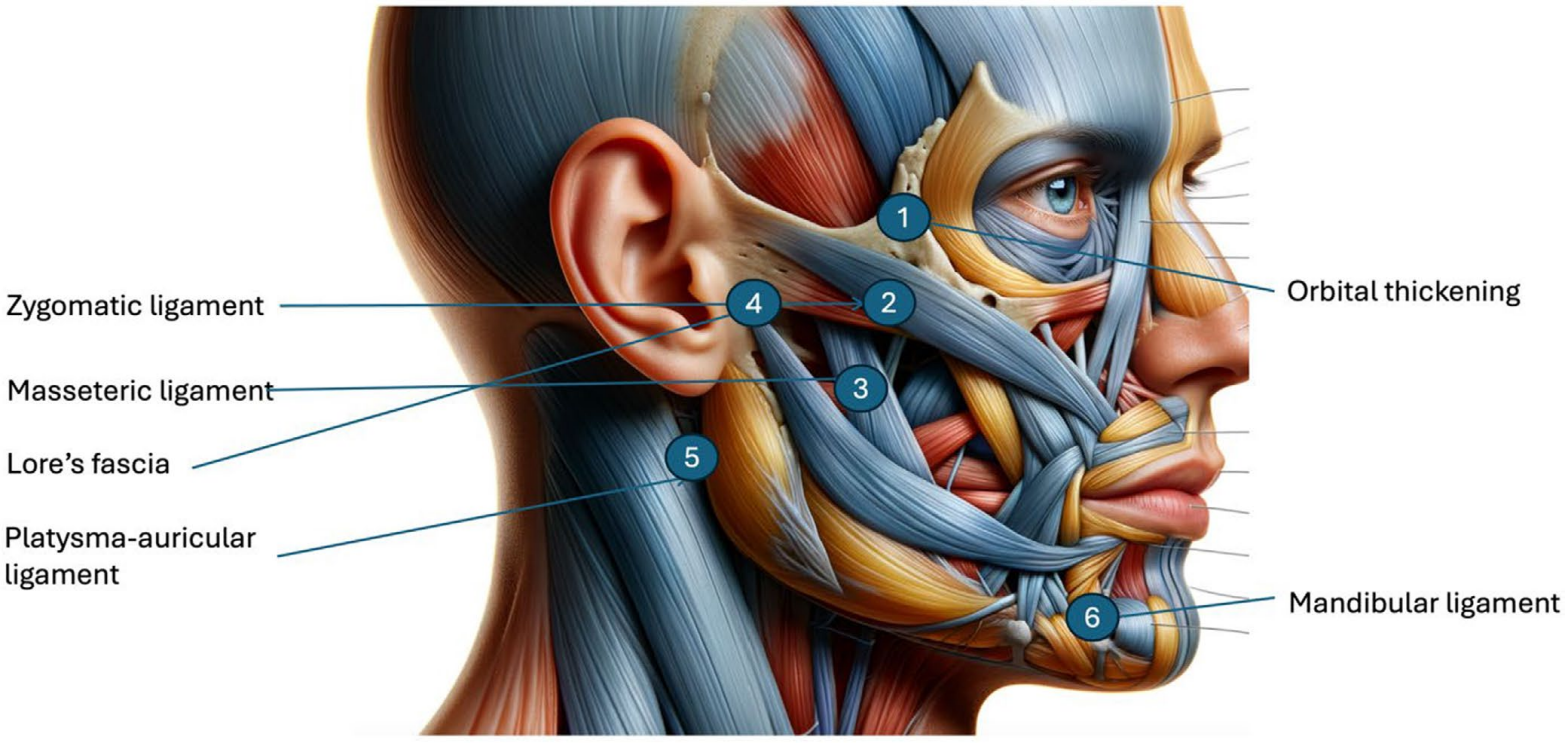
Injection points.

The injectable gel was administered by a licensed dermatologist. Each participant received three monthly sessions of deep injections into the subcutaneous layers of the facial skin, targeting the retaining ligaments. The procedure was performed under aseptic conditions using a 27G needle. Injection sites are shown in Figure 1.

The gel was injected deeply into the periosteum layer in the orbital thickening, as well as in the zygomatic cutaneous ligament and mandibular cutaneous ligament. For the other three points, they were placed in ligaments and fascia that lack underlying bone. In these cases, the injections were performed directly into the ligaments (upper masseteric ligament) and fascia (Lore's fascia and platysma‐auricular fascia) at a depth that varies among individuals, averaging 3–5 mm depending on the thickness of the dermis and subcutaneous tissue over the ligament or fascia.

## 3 Outcome Measures

### 3.1 Subjective Assessments

- *Global Aesthetic Improvement Scale* (*GAIS*): Both patients and investigators assessed aesthetic improvements at baseline (T0), 1 month (T1), 2 months (T2), 3 months (T3), and final follow‐up 3 months (T4) post‐treatment.

### 3.2 Objective Assessments

- *3D Photosystem Analysis*: Wrinkle depth and length were quantified using a 3D imaging system (LifeViz Mini, Quantificare, USA) and specialized software. A 3D photosystem provides comparable patient pictures with anatomical reference points, ensuring standardized and consistent images across multiple visits.
- *Lifting Vector Measurement*: A dedicated software tool (3D Track, Quantificare, USA) based on 3D analysis technology enable accurate measurement of straight and curve lines. It was used to measure the lifting effect of the gel, calculated as the average traction vector length between T0 and T4.

### 3.3 Statistical Analysis

Descriptive statistics were used to summarize baseline characteristics and outcome measures. Changes in GAIS scores and wrinkle depth were analyzed using repeated *t*‐test. A *p* < 0.01 was considered statistically significant.

### 3.4 Safety Assessments

Adverse events were recorded throughout the study period.

## 4 Results

The completion of this study by all participants serves as a proof of the tolerability and perceived efficacy of the novel injectable gel. Notably, no significant adverse effects were recorded, save for transient mild swelling at the sites of injection. The high viscosity, while necessitating a larger gauge needle, did not detract from the patient comfort, aligning with the findings of Flynn et al. [20] that gauge size may not significantly impact patient discomfort during facial injectable treatments.

### 4.1 Subjective Evaluation

**FIGURE 2:**
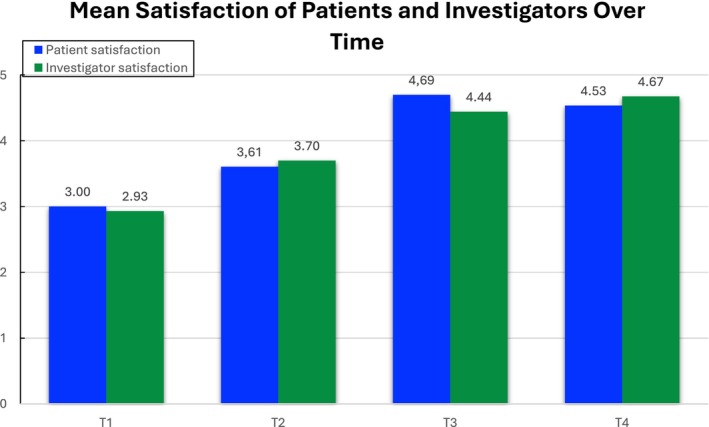
GAIS questionnaire (1—worsened, 2—unaltered, 3—improved, 4—very improved, 5—exceptional improvement).

Subjective assessment was made using GAIS questionnaire painted a promising picture of the injectable's performance, with participants (patients and investigator) reporting visible improvements in skin appearance following the initial treatment (Figure 2).

The graph above displays the average satisfaction scores over time for both patients and investigators at four different time points (T1, T2, T3, T4). The blue line represents patient satisfaction, while the green line shows investigator satisfaction. At time point T1(1 month after the first treatment), both scores start at a relatively lower level (around 3.0 for investigators and slightly higher for patients). This implies that the full effects of the treatment were not fully visible. By T2, there is a significant increase in satisfaction scores, with both curves steepening. This likely reflects visible improvements as the treatment effects become more apparent. Throughout T2 and T3, patient and investigator satisfaction scores remain relatively close, suggesting a consensus in the evaluation of aesthetic improvements. At T4 (3 months after the last treatment), both scores are above 4.5, which is still notably high, indicating overall success from both perspectives. This perceived enhancement in skin quality aligns with the intrinsic properties of HA to provide immediate improvements in skin hydration. The ongoing nature of these improvements, persisting 3 months post‐treatment, suggests a lasting effect, potentially attributable to ongoing collagen remodeling facilitated by the amino acids and polypeptides within the formula.

The paired *t*‐test was used to determine the statistical significance of the differences in satisfaction scores from T1 to T4. It showed very low *p*‐values (< 0.001), which are far below the common significance level threshold of 0.05. This indicates that the differences observed are highly unlikely to be due to random chance.

### 4.2 Objective Evaluation of Wrinkle Condition

**FIGURE 3:**
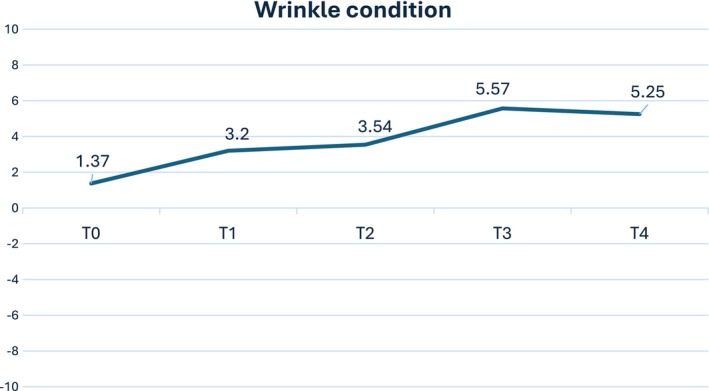
Standard deviation score improvement from baseline (T0) to final follow‐up (T4).

The objective evaluation was conducted using a 3D Photosystem and specialized software that calculates the depth and length of wrinkles. Standard deviation scores compare individual measurements to the mean of a reference population by expressing how many standard deviations an individual's measurement is from the mean. So, each patient's wrinkle condition was measured. The patient's score was compared to the mean of the reference population (Figure 3).

As clearly observed (Figure 3), the wrinkle condition starts at a score of 1.37, which is slightly better than the average score of the general population (T0). This represents the baseline measurement at the beginning of the observation period. After the first session of the injections the wrinkle condition score improves from 1.37 to 3.2 (T1). This indicates a noticeable improvement in wrinkle condition during this interval. The score continues to improve, though at a slower rate, rising from 3.2 to 3.54 from T1 to T2. From T2 to T3 there is a significant improvement in the wrinkle condition score from 3.54 to 5.57. This sharp rise suggests a substantial enhancement in the wrinkle condition during this period. The score slightly decreases from 5.57 to 5.25 at the end of the observational period (T4). This indicates a slight worsening or stabilization in wrinkle condition after the previous significant improvement. This trend highlights the overall enhancement in wrinkle condition over time from T0 to T3, with the most significant improvement observed between T2 and T3. The slight decrease from T3 to T4 might indicate a need for maintenance or additional treatment to sustain the improvement.

The statistical tests have been performed, and the results indicate that the differences in wrinkle conditions from T0 to T4 are statistically significant for both sides (*p* < 0.01).

### 4.3 Objective Evaluation of Lifting Effect

The objective measurement of the lifting effect in the study was meticulously conducted using advanced 3D Photosystem analysis and a dedicated software tool designed for precise measurement of lifting vectors. This innovative approach allowed us to quantitatively assess the changes in the skin's structural integrity and firmness over time, providing a reliable metric for evaluating the efficacy of our injectable gel.

**FIGURE 4:**
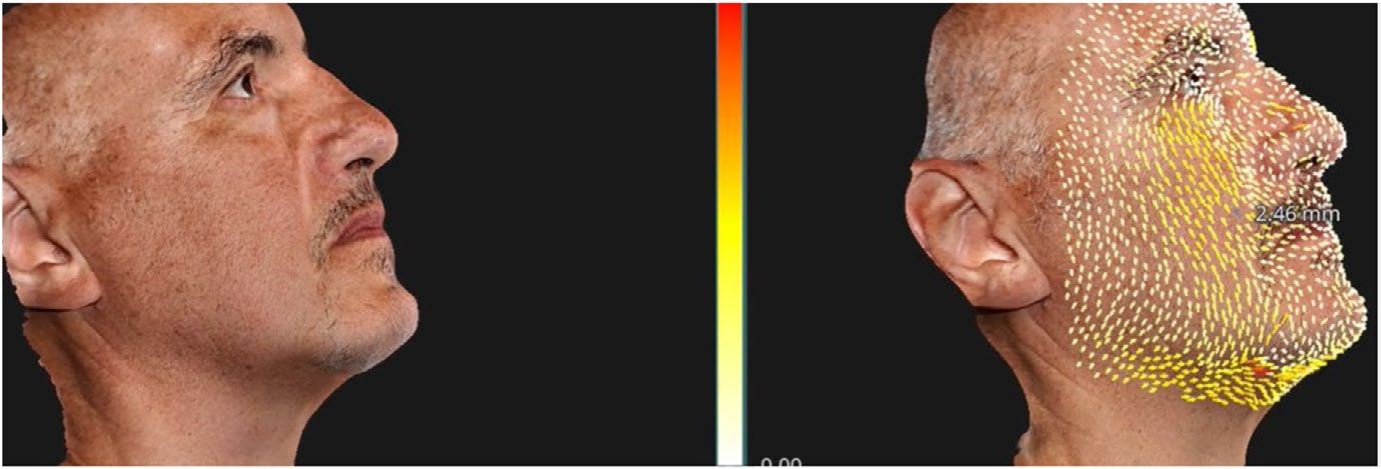
Lifting vectors in Patient 1.

**FIGURE 5:**
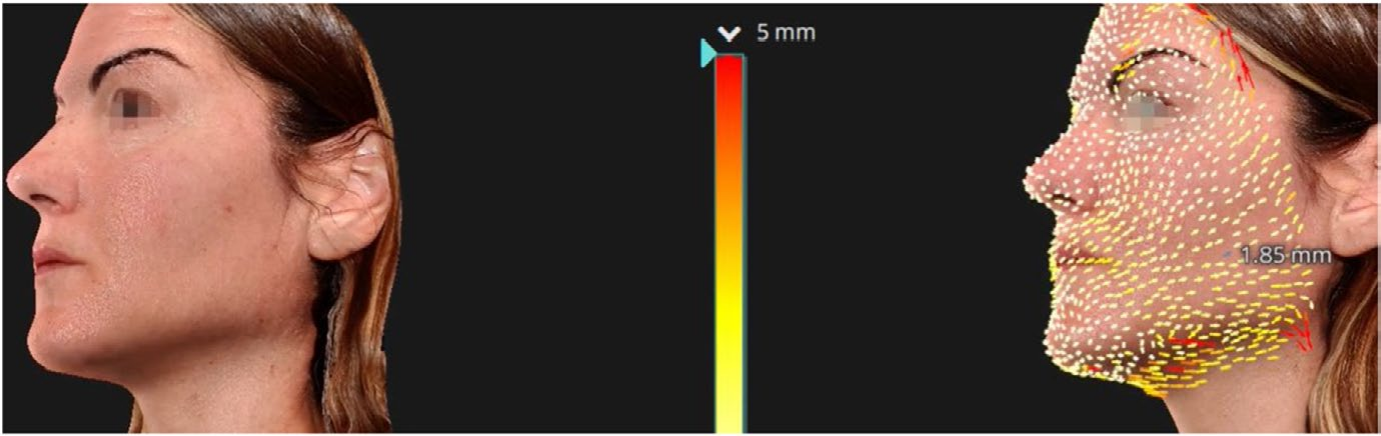
Lifting vectors in Patient 2.

**FIGURE 6:**
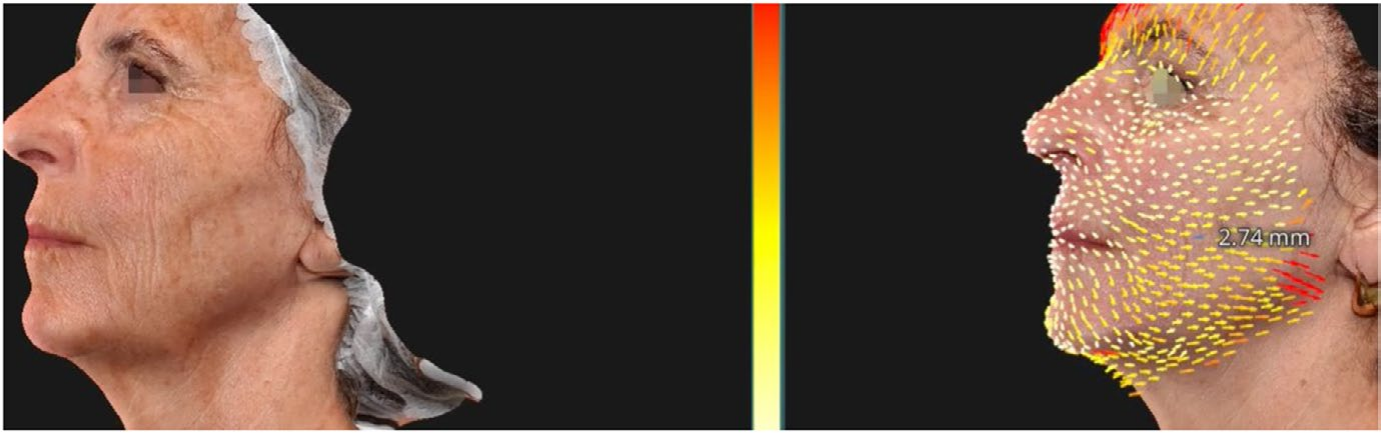
Lifting vectors in Patient 3.

During the course of the study, we observed a significant improvement in the lifting effect, with an average traction vector length increase of 1.6 mm measured between the baseline (T0) and the final follow‐up (T4). This metric quantifies the tightening and firming capacity of the gel, indicating a substantial enhancement in the skin's resilience and structural lift (Figures 4, 5, 6).

The lifting effect was statistically significant, with *p* < 0.01 indicating that the observed improvements were highly unlikely to be due to chance. These results are consistent with recent studies such as those by Kapoor et al., which demonstrate the potential of similar injectable treatments to significantly improve facial contours and skin firmness through targeted application [19].

## 5 Discussion

The treatment schedule employed in this study, consisting of three injections administered at four‐week intervals, was specifically designed to optimize the biostimulatory effects of the gel formulation. Unlike volumizing fillers, which primarily aim to restore volume through immediate mechanical filling, the studied product functions as a biostimulator. Its effectiveness lies in promoting collagen production and improving skin architecture over time.

The gel's unique composition, featuring both low and high molecular weight HA along with bioactive peptides, underpins the rationale for this phased approach. High molecular weight HA contributes to structural support and hydration, while low molecular weight HA facilitates deeper penetration and stimulates angiogenesis, collagen synthesis, and tissue repair. Bioactive peptides, acting as growth factor analogs, further amplify fibroblast activity and extracellular matrix remodeling.

Administering the injections over multiple sessions ensures sustained stimulation of these processes, allowing cumulative effects on tissue regeneration. This approach is supported by prior studies on biostimulators, which highlight the benefits of repeated cycles in achieving optimal outcomes [11, 19]. The schedule also reflects practical clinical considerations, allowing sufficient time for the skin's natural reparative processes between sessions.

This study's comprehensive analysis combining subjective and objective evaluations revealed significant improvements in both wrinkle conditions and the lifting effect. Subjectively, the GAIS recorded consistent enhancement in patient and investigator satisfaction over time, with final scores reflecting a high degree of aesthetic improvement. Objectively, 3D Photosystem analysis demonstrated notable decreases in wrinkle depth, corroborating the subjective assessments.

This study's findings emphasize the significant lifting effects achievable through ligaments injection technique. These outcomes align with the effectiveness of HA in facial rejuvenation, as demonstrated by Kapoor et al. [19], but with a specialized approach involving amino acids, and polypeptides. This suggests that deeper structural targeting, coupled with our gel's specific properties, might offer superior aesthetic improvements.

The correlation between injection depth and observed lifting effects underscores the potential advantages of this technique over more superficial treatments, supporting the trend towards minimally invasive procedures that deliver natural‐looking results and minimal downtime.

Moreover, the methodology employed in this study aligns with recommended practices in aesthetic measurement, ensuring that the lifting effects are not only perceptible to patients and clinicians but also statistically validated. We used a combination of subjective assessments (Global Aesthetic Improvement Scale) and objective measurements (3D Photosystem analysis) to capture a comprehensive view of the treatment's impact.

The robust lifting effects observed underscore the capability of the novel gel formulation to deliver targeted, deep‐tissue rejuvenation, effectively countering the gravitational and structural decline that characterizes facial aging. The use of high and low molecular weight HA gel, combined with a precise blend of amino acids and polypeptides, ensures that the lifting effect is both immediate and enduring, offering patients visible and lasting improvement.

During the study, we observed a significant improvement in the lifting improvement in skin firmness, contour, and support. In surgical facelifts, this effect is achieved through physical tightening and repositioning of skin and underlying tissues to counteract sagging.

In contrast, “lifting effect” in aesthetic medicine seeks to replicate aspects of this effect by using products and techniques that enhance structural support and elasticity in the skin and subcutaneous layers, without invasive intervention.

In this study, the “lifting effect” refers specifically to the measurable increase in skin firmness, structural support, and contour enhancement observed following the deep injections of the studied product. This effect was quantified using a 3D photosystem, which tracked changes in the “traction vector length” as a key metric for skin lifting.

Data from the study indicated a consistent increase in this vector length across the treatment timeline, averaging a 1.6 mm improvement between baseline (T0) and final follow‐up (T4).

Unlike a surgical lift, which repositions tissue immediately, this injectable formulation works through gradual collagen regeneration and tissue support, as seen by the sustained improvement in skin firmness over 3 months post‐treatment.

Thus, while non‐surgical, the 1.6 mm lift achieved in this study effectively represents a lifting effect due to the combined benefits of HA, amino acids, and polypeptides in the formula.

While this study did not specifically isolate the role of amino acids and polypeptides in collagen synthesis, there is substantial evidence in the literature supporting their efficacy in this regard. For instance, studies have shown that amino acids like proline and glycine are critical building blocks for collagen, directly supporting collagen production and improving skin elasticity and firmness [21]. Polypeptides have been shown to regulate extracellular matrix remodeling and reduce inflammation, which promotes a more stable and healthy collagen structure [22]. Additionally, bioactive collagen peptides have been reported to upregulate extracellular matrix synthesis, leading to increased skin firmness and resilience [23]. These findings reinforce the beneficial impact of the components used in our formulation.

Recent evidence further supports this mechanism. Shelemba et al. demonstrated that an injectable solution containing HA, amino acids, and peptides significantly increased collagen density in the dermis and subcutaneous connective tissues, including the retinacular cutis. High‐resolution ultrasound imaging revealed a 20.27% increase in collagen density 30 days after treatment, with a sustained 16.71% increase at 120 days. These findings highlight the ability of such bioactive formulations to stimulate neocollagenesis, even in mature skin where collagen turnover is inherently slow. While our study did not employ direct imaging of collagen density, the observed lifting effects and improvements in skin elasticity align with these results, suggesting similar regenerative processes were likely at play [24].

The significant increase in the standard deviation score for wrinkle conditions further quantifies the gel's efficacy, implying a substantial uniformity in the anti‐aging effects experienced by the study group. The convergence of subjective satisfaction and objective clinical improvements underscores the potential of the injectable formula as a formidable player in the realm of non‐invasive aesthetic medicine. By directly addressing the underlying structural components of the skin and facial architecture, this study contributes to the evolving landscape of cosmetic dermatology, wherein patient comfort, safety, and satisfaction are paramount.

## 6 Conclusion

The conclusions drawn from the study findings affirm the safety and efficacy of the injectable formula composed of essential amino acids, polypeptides, and high molecular weight HA, for non‐invasive skin rejuvenation treatments with target on lifting effect. These results are consistent with the growing body of evidence supporting the use of injectable formulations as viable alternatives to traditional invasive procedures for addressing age‐related skin concerns. The observed high patient satisfaction underlines the acceptability and desirability of the injectable formula among individuals seeking aesthetic enhancements. This positive feedback is indicative of the formulation's ability to deliver tangible improvements in skin appearance and overall satisfaction with treatment outcomes.

Moreover, the significant improvements in wrinkle conditions observed in the study provide objective evidence of the injectable formula's ability to target and address specific signs of aging, such as fine lines, wrinkles, and skin laxity. These findings are consistent with the proposed mechanisms of action of the formulation, which include promoting collagen regeneration at deep level, enhancing skin hydration, and stimulating cellular turnover; following the observation of Tsukahara et al. showing the relation between density of the retinacula cutis and depth of wrinkles [25].

The noticeable lifting effects reported by patients further validate the formulation's efficacy in promoting skin firmness and resilience, key attributes of youthful skin.

## 7 Limitations of the Study

Although the study offers encouraging results concerning the effectiveness and safety of a new injectable gel for non‐invasive skin rejuvenation, there are some notable limitations. The study has a relatively small sample size, suggesting that larger studies are necessary to validate these findings. Additionally, the lack of a control group and the failure to consider potential confounding factors such as participants' lifestyle habits, skincare routines, or sun exposure history could affect the reliability of the outcomes. Moreover, while the observed improvements in wrinkle conditions and lifting vectors in the study indirectly suggest tissue remodeling through collagen synthesis, further studies involving biochemical assays or histological evidence are necessary to confirm collagen production.

## Ethics Statement

All procedures performed in this study involving human participants were in accordance with the ethical standards of the institutional and national research committee and with the 1964 Helsinki declaration and its later amendments or comparable ethical standards. Informed consent was obtained from all individual participants included in the study.

## Consent

Informed consent was obtained from all individual participants for whom identifying information is included in this article. Participants provided their consent for the use of their photographs for publication purposes.

## Conflicts of Interest

The author declares no conflicts of interest. The product used in this investigation was provided by Professional Derma SA.

## Data Availability

The data that support the findings of this study are available from the corresponding author upon reasonable request.
